# Pain Mechanism in Rheumatoid Arthritis: From Cytokines to Central Sensitization

**DOI:** 10.1155/2020/2076328

**Published:** 2020-09-12

**Authors:** Yanting Cao, Danping Fan, Yiqing Yin

**Affiliations:** ^1^Department of Anesthesiology, China-Japan Friendship Hospital, Beijing, China; ^2^Institute of Clinical Medicine, China-Japan Friendship Hospital, Beijing, China; ^3^Graduate School of Peking Union Medical College, Chinese Academy of Medical Sciences/Peking Union Medical College, Beijing, China; ^4^Department of Anesthesiology, Tianjin Medical University Cancer Institute and Hospital, Tianjin, China

## Abstract

Pain is the most common symptom in patients with rheumatoid arthritis (RA). Although in recent years, through the implementation of targeted treatment and the introduction of disease-modifying antirheumatic drugs (DMARDs), the treatment of RA patients has made a significant progress, a large proportion of patients still feel pain. Finding appropriate treatment to alleviate the pain is very important for RA patients. Current research showed that, in addition to inflammation, RA pain involves peripheral sensitization and abnormalities in the central nervous system (CNS) pain regulatory mechanisms. This review summarized the literature on pain mechanisms of RA published in recent years. A better understanding of pain mechanisms will help to develop new analgesic targets and deploy new and existing therapies.

## 1. Introduction

Rheumatoid arthritis (RA) is one of the most common types of arthritis, with the prevalence of 0.3% to 4.2% in different populations [1, 2]. The most common symptom of RA is pain. 97% of early RA patients feel pain, which is also the main reason for early RA patients to visit a healthcare professional. 90.4% of early RA patients visit a healthcare professional because of intense pain [3]. Pain starts before the manifestations of RA [4, 5], which will cause psychological distress and sleep disorders [6]. And pain is considered as an important factor affecting valuable life activities. Even if the pain level is low, it will also restrict the activity [7]. Moreover, it is also a determinant of the patients' assessment of disease activity [8]. However, for doctors, the contribution of pain to the overall evaluation of the disease is relatively small, while joint swelling is the most important factor for doctors to evaluate the disease activity [8]. Although the treatment of RA has made remarkable progresses in recent years, which has greatly improved the control of disease activity and joint damage, the degree of pain in RA patients included in the 2000s did not alleviate compared with that included in the 1990s over 8 years of follow-up [9]. From December 2017 to January 2018, a cross-sectional survey of RA patients was conducted in the United States. The patients have failed ≥1 DMARDs and were receiving current DMARD(s) for ≥6 months. Only 26% of the patients were satisfied with RA treatment. Patients who were not satisfied with the treatment had more severe pain. The study also shows that pain is one of the most important symptoms of RA affecting the life of RA patients. Even patients who were satisfied with the treatment tended to report moderate pain symptom severity [10]. These studies have shown the importance of pain to RA patients and indicated that doctors should pay more attention to RA pain.

There is no doubt about the contribution of inflammation to RA pain, but research shows that there is a discrepancy between doctors' assessment of inflammation and patients' reported pain, with 64% of participants in pain flare not concurrently in DAS28 flare and 60% of those in DAS28 flare not concurrently in pain flare [11]. During acute synovitis, the intensity of pain is related to the severity of inflammation, but the association between pain and joint inflammation is often weak before disease flaring and after the suppression of inflammatory disease [5, 12–17]. The prevalence of clinically significant pain is 11.9% among patients meeting the Disease Activity Score (DAS28) remission criteria. Among patients in DAS28 remission, inflammatory disease activity is not significantly associated with elevated pain severity [13]. At this time, noninflammatory mechanism may be the main cause of RA pain. In patients with pain mainly caused by factors other than inflammation, intensive DMARD treatment may not only be ineffective but also unnecessarily expose people to the risk of adverse events, leading to unnecessary treatment changes [18, 19]. Therefore, there is an urgent need to improve our understanding of the pain mechanism of RA to help develop new treatment strategies.

**Figure 1:**
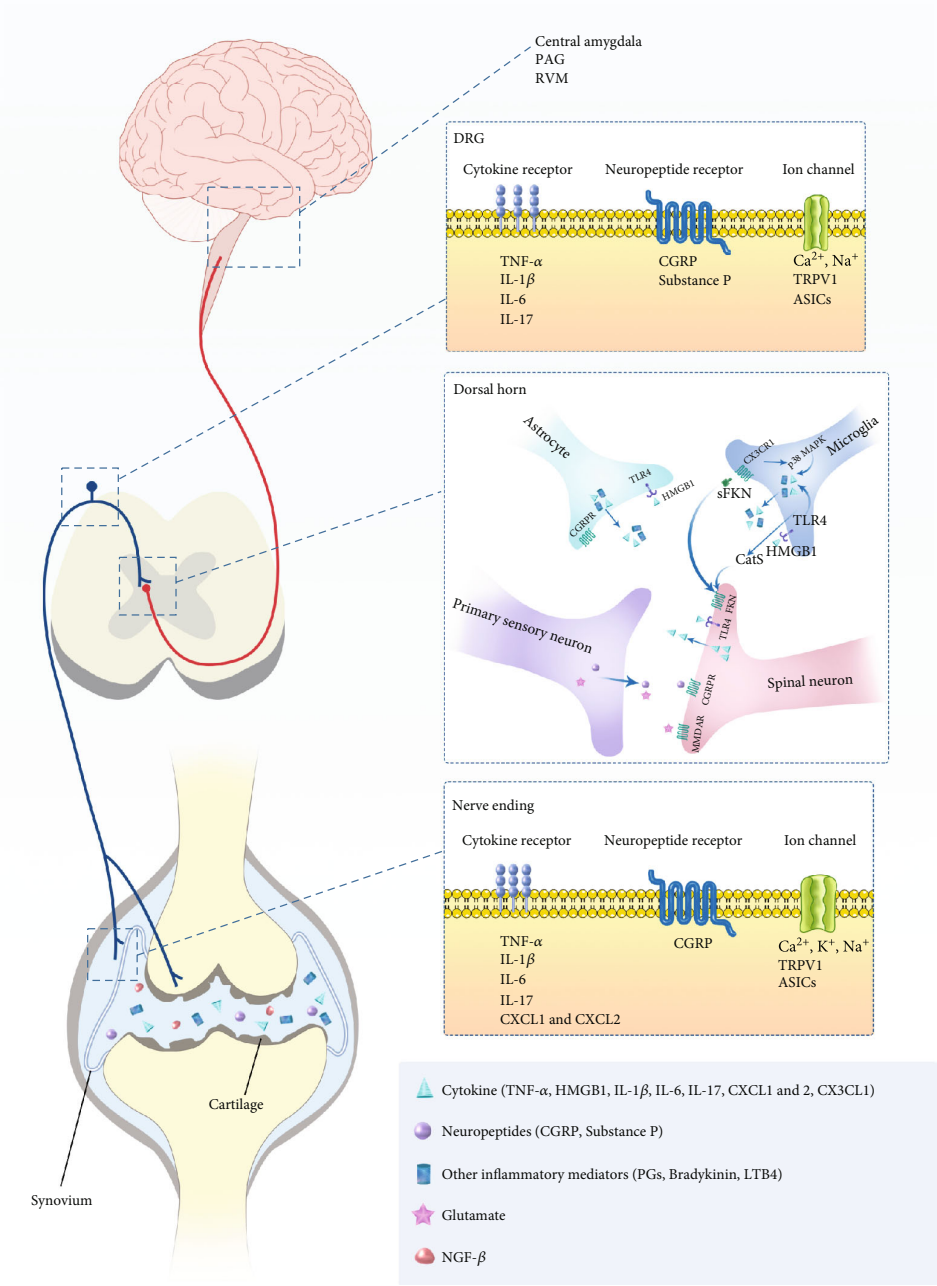
Schematic overview of reported peripheral and central mechanisms of RA pain. Most of the structures in the joint are dominated by nociceptive neurons whose cell bodies are located in the DRG. During joint inflammation, the resident cells and infiltrating immune cells in the joint release proinflammatory cytokines (e.g., TNF-*α*, IL-1*β*, IL-6, and IL-17) and the sensory nerve endings release CGRP, which activate and/or sensitize the primary afferents. The activation of primary afferents results in the change of DRG gene expression. Glutamate and neuropeptides (e.g., CGRP) are released from central nerve endings and peripheral sensory nerve endings, and their receptors are activated, respectively. Activated astrocytes and microglia release proinflammatory cytokines (e.g., TNF-*α*) in the spinal cord, which will contribute to the spinal sensitization. Microglia also release CatS, cutting FKN on spinal cord neurons, and the resulting soluble FKN (sFKN) further enhances the reactivity of microglia via CX3CR1. HMGB1 is released from spinal neurons and activates TLR4 on glial cells and spinal neurons. The increase of CGRP in the dorsal horn regulates the secondary afferent activity. CGRP is widely expressed in the central nervous system (CNS) and plays a role in the amygdala, pons, and other brain stem areas to enhance the nociceptive signal. DRG: dorsal root ganglion; TNF-*α*: tumor necrosis factor-*α*; IL-1*β*: interleukin-1*β*; IL-6: interleukin-6; IL-17: interleukin-17; CGRP: calcitonin gene-related peptide; CatS: cathepsin S; FKN: fractalkine; HMGB1: high-mobility group box 1 protein; TLR4: toll-like receptors; NGF-*β*: nerve growth factor-*β*.

RA pain results from the interaction of joint pathology with peripheral, spinal, and supraspinal pain signaling. The intensity, distribution, and characteristics of RA pain depend on the direct activation of peripheral pain receptors and the regulation of neuronal sensitivity in the whole pain signaling pathway (Figure 1) [20].

## 2. Nociceptive Pain and Peripheral Sensitization

The synovium and capsule of the joint are mainly dominated by the peripheral afferent fibers of the dorsal root ganglion (DRG). In these areas, there are a large number of primary A*α* and A*β* sensory neurons involved in mechanosensation and A*δ* and C fibers involved in nociception [21]. Synovitis is the main pathophysiological mechanism of RA, which can directly activate and sensitize the afferent nerve [18, 22].

Bradykinin and prostaglandins are increased in synovial fluids from RA patients which can directly activate the sensory nerve endings within the synovium [23]. Synovial fluid concentrations of tumor necrosis factor-*α* (TNF-*α*), interleukin-1*β* (IL-1*β*), interleukin-6 (IL-6), interleukin-17 (IL-17) [24, 25], calcitonin gene-related peptide (CGRP) [26, 27], and nerve growth factor-*β* (NGF-*β*) [28] are increased in RA patients, which can directly alter the responses of nociceptive neurons [12].

The synovium is not the only source of RA pain. Moreover, there are also sensory nerves in the joint capsule, lateral area of meniscus, subchondral bone, ligament, tendon sheath, and muscle, which will also play an important role in the generation of chronic pain [23]. The inner two-thirds of the menisci and articular cartilage are normally aneural. It has been confirmed that inflammation and production of cytokines and NGF-*β* are present in subchondral bone of RA patients [29]. Subchondral erosion destroys osteochondral connections, which may expose subchondral nerves to activating and sensitizing factors in synovial fluid. These pathological innervations can further aggravate the pain of weight bearing or joint movement.

## 3. CNS Regulation Mechanisms

In the absence of persistent local inflammation or signs of local tissue destruction, the pain threshold for adjacent tissues of joint in RA is reduced and may extend to the cephalic, caudal, and opposite parts of the affected joint. The wide distribution of hyperalgesia suggests that persistent pain may be caused by central pain regulation mechanisms rather than peripheral stimulation of nociceptors [30]. According to the central sensitization inventory (CSI), 41% of RA patients had central sensitization syndrome [31]. Before the clinical characteristics of arthritis become obvious, the central pain processing has changed [32, 33]. Lower pressure pain thresholds in patients with a longer history suggest that the central-mediated mechanisms develop over time [34].

The changes of central pain mechanisms include (1) lack of descending inhibition pathways; (2) enhancement of descending facilitatory pathways; and (3) central sensitization at the spinal cord level. In the descending pathway of pain, the periaqueductal gray (PAG) and rostral ventromedial medulla (RVM) are important regulatory centers. The PAG receives input, such as mood and stress, from the frontal cortex, amygdala and hypothalamus, which will affect pain perception [25, 35]. The PAG integrates these signals and transmits them to the RVM. Depending on the specific pathway of activation, the RVM can facilitate and/or inhibit pain. Serotonin and norepinephrine are important neurotransmitters in the descending pain regulation pathway [35]. They can regulate the excitability of spinal dorsal horn neurons and the release of nociceptive afferent neurotransmitters by binding with different receptors. Central sensitization at the spinal level occurs in the dorsal horn of the spinal cord, leading to the enlargement of the receptive field and the enhancement of pain sensitivity. Two primary phases exist as follows: (1) the acute stage mediated by the binding of glutamate released by primary afferent neurons with N-methyl-D-aspartate (NMDA) receptor, which is distributed on the postsynaptic neurons of the spinal dorsal horn, and (2) the chronic stage mediated by the activation of spinal microglia and the transcription of pain-regulating peptide [36]. Synovitis is associated with the increase of substance P, CGRP, and their receptors in the spinal cord [23]. Glutamate binds to postsynaptic NMDAR receptor; CGRP acts on postsynaptic CGRP1 receptor; substance P binds to neurokinin-1 (NK1) G-protein-coupled receptor, and finally, signal pathways such as PKA and PKC are activated and lead to central sensitization [36]. CGRP is widely expressed in the CNS and plays a role in the amygdala and other brain stem areas to enhance the nociceptive signal [37]. The microglia and astrocytes in the spinal cord are activated after the peripheral nervous system is stimulated by injury and enter a state of enhanced response related to pain, producing cytokines such as TNF-*α*, interleukin-1 (IL-1), and IL-6 [24, 38]. Spinal cord exposure to TNF-*α*, IL-1*β*, and IL-6 can lead to hyperalgesia and allodynia [12, 32, 39–41].

Recent studies have shown that neuropathic-like symptoms in rheumatic conditions are the manifestation of central pain management mechanism disorder [17]. Neuropathic pain (NP) is believed to be caused by diseases or lesions affecting the somatosensory nervous system [42], which can persist without noxious stimulation and peripheral inflammation [43]. Although RA pain is often described as “aching” or “gnawing” which is typically associated with nociceptive pain, some RA patients also have typical NP characteristics such as “prickling” or “burning” [17]. Increasing evidence has demonstrated that pain for a substantial number of RA patients may have a neuropathic component or neuropathic features. Ahmed et al. reported that 5% of RA patients had features of likely NP and 28% had possible NP according to painDETECT [44]. In the study by Perrot et al., the proportion of patients with NP is relatively high (35.7%) [45], which may be due to their use of the DN4 questionnaire. The DN4 questionnaire has high sensitivity but low specificity in identifying NP [46]. Koop et al. [17] studied that 159 RA outpatients were screened for NP with the painDETECT screening tool and found that 17.0% of the patients were classified as likely suffering NP and 21.4% as having possible NP. These patients reported more severe pain and were more likely to have more tender joints and use analgesics. After multivariate analysis, NP features in RA patients are independently associated with self-reported mental and physical health [17]. For patients with RA who still cannot achieve remission after intensive anti-inflammatory treatment, pain treatment targeting the NP-like symptoms can be considered. This treatment can comprise certain antidepressants and anticonvulsants [47].

The study of the central pain processing in RA is still in its infancy. At present, there is no objective measurement for central sensitization. Its diagnosis is usually based on the combination of clinical or expert opinions and quantitative sensory test. The cytokine candidates that affect the central pain processing may not be those that have been traditionally developed as targets for the treatment of synovitis and may find new regulators [12]. Finding the mechanism of central sensitization and preventing its development have great potential for reducing the long-term pain burden of RA patients.

## 4. Cytokines and Chemokines

### 4.1. TNF-*α*

TNF receptor 1 (TNFR1) and TNF receptor 2 (TNFR2) can be detected in sensory neurons [20]. Injection of TNF-*α* into the knee joint of rats can enhance the response of spinal neurons to mechanical joint stimulation, while intra-articular injection of etanercept can reduce the spinal cord activity induced by inflammation [39]. After intra-articular injection of TNF-*α*, the response of C fiber to noxious and innocuous rotation of the joint shows a persistent increase in a dose-dependent manner, while the response of A*δ* fiber to noxious rotation is slightly increased. TNF-*α* can enhance the excitability and tetrodotoxin- (TTX-) resistant Na^+^ current of cultured DRG neurons in a few minutes [48, 49]. The increase of Na^+^ current depends on the activation of TNFR1 and p38 mitogen-activated protein kinase (MAPK) [49]. P38 inhibitor can prevent the mechanical sensitization of TNF-*α* [48, 49]. These studies indicated that TNF-*α* can induce the long-term sensitivity of nociceptors to mechanical stimulation by acting directly on TNFR1 in primary afferent neurons, thus inducing the long-term mechanical hyperalgesia of joints. As a sensitizing pain mediator, tumor necrosis factor can directly act on primary afferent neurons.

The application of TNF-*α* in the spinal cord of normal rats increased the response of the spinal cord to joint stimulation. In AIA rats, spinal response can be reduced by the intrathecal application of etanercept on day 1, but not on day 3 [39]. These suggested that TNF-*α* also modulates pain signals at the spinal cord level. This may be due to the direct effect of TNF-*α* on neurons or the indirect effect of TNF-*α* to activate microglia and release other cytokines. This also suggests that the established excitability of the spinal cord can be maintained by the downstream mechanism independently of spinal TNF-*α*. The role of TNF-*α* (and of other cytokines) in RA pain is summarized in Table 1.

### 4.2. IL-1*β*

The expression of IL-1R in the DRGs of AIA models and in the periarticular tissue of male collagen-induced arthritis (CIA) rats is significantly upregulated [50]. IL-1*β* makes the C fibers of joints sensitive to mechanical stimulation. However, the sensitivity of A*δ* fiber is also significantly reduced by IL-1*β*. The effect of IL-1*β* on the pain of arthritis is an interesting problem because A*δ* and C fiber gather on the same spinal cord neurons [24]. The research showed that in the AIA model subcutaneous injection of anakinra, an IL-1*β* receptor antagonist, can reduce the thermal hypersensitivity but not the mechanical hypersensitivity [51, 52]. Because the main burden of RA is mechanical hyperalgesia rather than thermal hyperalgesia, anakinra may not cause pain relief in RA.

The expression of IL-1*β* gene in the spinal cord of male K/BxN rats is increased [53]. The levels of IL-1*β* in cerebrospinal fluid (CSF) of RA patients [53] and female CIA rats [32] are increased. And the concentration of IL-1*β* in CSF of RA patients was higher than that of serum, indicating local production in the CNS [53]. Intrathecal injection of IL-1*β* can increase responses of neurons in the rat dorsal horn. Intrathecal injection of IL-1*β* can also cause mechanical hyperalgesia and hyperalgesia [41]. Intrathecal application of microglial inhibitors (P2X7 antagonists) can reduce the level of IL-1*β*, microglial proliferation, and mechanical hypersensitivity in CIA female rats [32].

Therefore, these results can explain the limited effect of the inhibition of IL-1*β* on RA-related pain and indicate that central IL-1*β* signal, rather than peripheral or systemic IL-1*β*, may be the target of RA pain treatment.

### 4.3. IL-6

The concentrations of IL-6 and soluble IL-6 receptor (sIL-6R) in serum, synovial fluid, and tissues of RA patients are increased. IL-6 receptor (IL-6R) contains two chains of glycoprotein 130 (gp130), which is the signal-transducing *β*-receptor subunit of IL-6 [24, 54].

Gp130 is expressed by neurons and glia cells of the spinal cord, as well as the DRG, so these cells are sensitive to the transsignalling of IL-6/sIL-6R [54]. The effect of IL-6 on satellite glial cells has not been well studied. In AIA mice, the specific absence of gp130 in sensory neurons can alleviate joint inflammation and pain-like behavior [55]. Injection of IL-6 or coinjection of IL-6 together with sIL-6R into a normal knee caused an increase in the responses of C fibers to mechanical stimuli whereas the responses of A*δ* fibers were not altered. Coadministration of soluble gp130 (sgp130) can prevent the sensitization induced by IL-6 and IL-6 plus sIL-6R. But sgp130 did not reverse the established enhanced mechanosensitivity [56]. The application of IL-6/sIL-6R in the knee joint or spinal cord of rats increased the response of spinal cord neurons to mechanical stimulation of the knee joint and other parts of the legs, expanded the size of sensory field of neurons, and showed the potential of IL-6-induced central sensitization. During the development of inflammation, intrathecal application of sgp130, which binds IL-6/sIL-6R complexes and thus prevents transsignalling, can reduce the hyperactivity of the spinal cord and relieve pain-like behavior without inhibiting joint inflammation. However, if the inflammation develops completely, the application of sgp130 in the spinal cord cannot reverse the established hyperactivity [40]. Local injection of sgp130 into joints can significantly reduce the primary mechanical hyperalgesia and joint damage in the acute phase of AIA arthritis compared with repeated systemic injection of sgp130, but neither can reduce the secondary hyperalgesia [57].

These studies showed that IL-6/sIL-6R in the joints and spinal cord, rather than IL-6/sIL-6R in circulation, is the cause of hyperalgesia. Secondly, the early neutralization of IL-6/sIL-6R is particularly successful in analgesia. Therefore, early neutralization of IL-6/sIL-6R at the site of joint inflammation seems to be an effective way to treat RA pain. In patients with active RA, the use of sirukumab, a monoclonal antibody against IL-6, showed a dose-dependent improvement in pain [58]. It is not clear whether this is due to the inhibition of inflammation or the direct effect of inhibition of IL-6. Further research is needed to understand whether the inhibition of IL-6 has additional advantages in pain relief, especially in patients with hyperalgesia.

### 4.4. IL-17

Proinflammatory cytokine IL-17A-F is released by T-helper 17 cells and plays an important role in adaptability and innate immune system [59]. In male AIA mice, the level of IL-17 was increased in periarticular tissues [60]. In the AIA model, IL-17A knockout mice showed less mechanical hyperalgesia than wild-type mice, but there was no difference in the degree of inflammation [61]. In the AIA mouse model, anti-IL-17 antibody slightly reduced swelling, but significantly reduced secondary mechanical hyperalgesia in the paw. IL-17A mainly sensitizes C fibers. But at a very low dose, IL-17A reduced the responses of A*δ* fibers [62]. Intra-articular injection of IL-17 in mice can increase the production of TNF-*α*, IL-1*β*, and CXCL1 and lead to mechanical hypersensitivity and neutrophil recruitment. The hypernociceptive effect of IL-17 can be reduced in TNFR1^−/−^ mice and can also be reduced by the pretreatment with anti-TNF antibody, IL-1 receptor antagonist, and CXCR1/2 antagonist [63]. These results suggested that IL-17 is a novel pronociceptive cytokine, whose effect depends on various proinflammatory mediators.

Due to the fact that sensory neurons in the DRG express all IL-17 receptor subtypes, IL-17 directly increases the activity of DRG neurons. In the isolated DRG neurons, IL-17A-F increases tetrodotoxin- (TTX-) resistant sodium currents [61], which are believed to be related to the sensitization of sensory nociceptive neurons caused by inflammation. IL-17 stimulates primary cultured DRG neurons, which will result in the upregulation of transient receptor potential vanilloid 4 (TRPV4). TRPV4 is considered as a candidate transducer of mechanical hyperalgesia [64]. In the isolated DRG neurons, IL-17A induces rapid phosphorylation of ERK and protein kinase B within 5 minutes. And IL-17A rapidly enhances the excitability of DRG neurons [62]. *In vitro*, IL-17 can promote the proliferation ability of astrocytes and the expression of inflammatory cytokines in astrocytes [65].

These data suggested that IL-17 is involved in mechanical hyperalgesia. However, secukinumab, an anti-IL-17A antibody, has no convincing clinical effect on RA so far [66]. Thus, the role of IL-17 in RA pain still needs further study.

### 4.5. Interleukin-22 (IL-22)

IL-22 is produced by activated T cells and NK cells and participates in innate and adaptive immune system responses [67]. It has a dual role in arthritis: protective effect before the onset of arthritis and pathogenic effect after the onset of arthritis [68]. However, the relationship between IL-22 and RA pain has not been well studied. The expression of IL-22 mRNA in the synovium of male AIA mice is increased, and IL-22 antibody can protect mice from mechanical hypersensitivity. The articular pain in IL-22^−/−^ AIA mice is also decreased. Further study showed that IL-22 plays a pathogenic role in the onset of AIA through a C-terminal caspase recruitment domain- (ASC-) dependent stimulation of IL-1*β* production [69]. The specific mechanism of IL-22 involved in pain is not clear and warrants intensive studies.

### 4.6. CXCL1 and CXCL2

CXCL1 and CXCL2 are typical neutrophil recruitment activators whose receptors are CXCR1 and CXCR2 [70]. In the ankle and knee joints of AIA mice, the levels of CXCL1 and CXCL2 are increased, and the inhibition of CXCR1 and CXCR2 can reduce the mechanical hypersensitivity of mice, the production of TNF-*α*, and the neutrophil recruitment by inhibiting neutrophil adhesion [71]. It was found that ACPA may directly induce mechanical and thermal hyperalgesia through the way separated from inflammation. This effect was related to ACPA-mediated release of CXCL1 and activation of osteoclasts. Rapamycin, a CXCR1/2 antagonist, can reduce pain-like response induced by ACPA [72]. CXCL1 increases the excitability and sensitization of nociceptors by activating neuronal CXCR2 [50].

CXCL1 and CXCL2 drive RA-related pain through indirect mechanisms such as neutrophil recruitment and through direct action on nociceptors. If these findings can be replicated, new analgesic targets may be found.

### 4.7. CX3CL1

CX3CL1, also known as fractalkine (FKN), is a chemokine whose receptor CX3CR1 is exclusively expressed in microglia [73]. Intrathecal application of FKN can activate CX3CR1 expressed by microglia, which will lead to p38 MAPK-mediated release of cytokines that can enhance pain signal transmission [20]. The mechanical hypersensitivity of the CIA model is closely related to the cathepsin S (CatS)/FKN signaling of reactive microglia. In animal models, CatS inhibitors prevent pain-like behavior and the development of spinal cord hyperactivity without any effect on joint inflammation [50]. In addition, intrathecal injection of FKN-neutralizing antibody and CatS inhibitor can reduce the mechanical hypersensitivity of CIA rats and inhibit the microglia reaction in the spinal cord but cannot prevent the development of arthritis [74]. CX3CR1 antagonist is a new way to treat RA pain.

## 5. Toll-Like Receptors (TLRs)

TLRs are expressed in immune cells, chondrocytes, synovial cells, osteoclasts, glial cells, and sensory neurons [75–77]. They are activated by endogenous damage-associated molecular patterns (DAMPs) [76]. DAMPs such as tenascin-c, heat-shock proteins, and high-mobility group box 1 protein (HMGB1) are increased in the RA model [50].

TLR4 is expressed in spinal microglia in abundance. When TLR4 is activated, a large number of cytokines, chemokines, and lipids are secreted. These products act on receptors of spinal dorsal horn neurons, enhance pain signaling, and produce hyperalgesia [78]. Blocking HMGB1-TLR4 signaling in the spinal cord of collagen antibody-induced arthritis (CAIA) mice can reverse the mechanical hypersensitivity in the inflammatory stage and late stage of the model [50]. Some studies suggested that TLR4 signaling in the spinal cord has little effect on acute pain state, but it seems to play a key role in the transition from acute mechanical hypersensitivity to chronic hypersensitivity [79, 80]. In the K/BxN mouse models, intrathecal injection of the TLR4 antagonist LPS-RS or TLR4 knockout had no effect on early allodynia but could prevent postinflammation allodynia [79]. After injection of formalin into the hind paw of mice, these animals showed tactile hypersensitivity and strong cringe behavior. Pretreatment with TLR4 antagonist (TAK-242) had no effect on the initial hypersensitivity, but the late paralgesia can be prevented [80]. It was found that TLR4-induced production of the bioactive lipids by 12/15-lipoxygenase (12/15-LO) partially mediates neural sensitization, although the contribution of 12/15-LO expressed in other cell types cannot be excluded [81]. In conclusion, these studies indicated that TLR mediates RA pain through direct or indirect mechanism.

## 6. Fc-Gamma Receptors (Fc*γ*Rs)

Fc*γ*Rs bind to the Fc domain of antibodies (IgG) and are activated by immune complexes (ICs) to regulate adaptive immunity. Compared with normal mice, the expression of Fc*γ*RI in the small-sized DRG neurons of female AIA rats is increased. The excitability of DRG neurons stimulated by IgG-IC is enhanced. Intradermal injection of IgG-IC can also increase the excitability of C fibers *in vivo* and produce mechanical hypersensitivity [82]. Anti-collagen type II (CII) antibodies in IC with CII induce inward current in primary DRG neurons of mouse, increase intracellular [Ca^2+^], and release pain-related CGRP. However, the expression of Fc*γ*RI on satellite cells is decreased and CII-IC fails to induce CGRP release in cultures generated from FcR*γ*-chain^−/−^ mice, but in cultures from Fc*γ*RIII^−/−^ mice, intracellular [Ca^2+^] signal is still increased. In FcR*γ*-chain^−/−^ mice, which lack all activated Fc*γ*Rs but still express Fc*γ*RIIb, the pronociceptive actions of cartilage-associated antibodies injected systemically and locally are reduced, and IC-evoked release of CGRP in primary neuronal DRG cultures is prevented. In addition, the lack of Fc*γ*RIII and Fc*γ*RIV does not decrease the intracellular [Ca^2+^] induced by CII-IC and pain-like behavior induced by CII antibody [83]. Therefore, Fc*γ*RI is responsible for mediating the role of cartilage-related antibodies in RA-related pain.

## 7. Leukotriene B4 (LTB4)

The content of LTB4 in the joint fluid of RA patients is high [4]. The synthesis of LTB4 mainly depends on 5-lipoxygenase (5-LO) and leukotriene A4 hydrolase (LTA4H). LTB4 initiates inflammatory signaling cascades by binding its high-affinity receptor BLT1, leading to the activation and recruitment of leukocytes. However, BLT2, the low-affinity receptor of LTB4, is rarely studied [84, 85]. In the AIA model, the incidence and severity of arthritis in BLT2^−/−^ mice decreased, and the destruction of bone and cartilage reduced [85]. Cortes-Burgos et al. reported that the concentration of LTB4 in the brain of RA rat model is three times higher than that of the control group. CJ-13610, a 5-LO inhibitor, can inhibit the synthesis of LTB4 and improve hyperalgesia, suggesting that LTB4 and 5-LO pathway are important mediators of pain [86].

## 8. Ion Channels

### 8.1. Calcium Channels

The level of the auxiliary subunit *α*-2*δ*-1 (*α*2*δ*1) of voltage-gated Ca^2+^ channels in DRG neurons is increased when arthritis subsides but pain still exists in male CAIA mice [87]. Gabapentin blocks Ca^2+^ flux by interacting with *α*2*δ*1 subunit [50]. It can reverse the mechanical hypersensitivity in the inflammatory phase and late stage of CAIA mice [88]. These findings demonstrated the importance of *α*2*δ*1 subunit in pain behaviors induced by arthritis. The inhibition of store-operated Ca^2+^ channels by oral administration of YM-58483 can prevent and reverse the mechanical hypersensitivity of male CIA mice and reduce joint inflammation [89].

These findings suggested that calcium channels are potential targets for pain management in arthritis. However, it has been found that in the transgenic mice that specifically block the voltage-gated calcium 2.2 (CaV 2.2) channel, the pain caused by arthritis is significantly reduced, but the mice showed persistent inflammation, upregulation of osteoclast activator RANKL, and destruction of the joint and bone [90]. It may not be likely to use CaV 2.2 channel blockers as analgesics during inflammation. The role of ion channels in RA pain is summarized in Table 2.

### 8.2. TRPV1

TRPV1, a nonselective cation channel, is expressed by nociceptors and activated by endogenous lipids, protons, and heat [50]. Neutralization of TRPV1 can reduce the mechanical hypersensitivity of male CIA rats [91]. TRPV1^−/−^ mice have reduced synovium inflammation, bone erosion, and cartilage damage from the second week, but weakened bilateral hyperalgesia until the eighth week, indicating that TRPV1 is involved in chronic RA pain [92].

However, the role of TRPV1 in RA-related pain is controversial. In clinical trials of RA pain, one drug strategy emphasizes inhibition of TRPV1, while another completely different method is activation of TRPV1 [93]. Several studies have found that TRPV1 antagonists do not reduce pain behavior in arthritis models [94]. Many inhibitors of TRPV1 have been developed and tested, but up to now, due to the lack of efficacy and other reasons, these compounds have not been approved for clinical use [93]. Capsaicin is the activator of TRPV1, and cntx-4975 is the *trans*-isomer of capsaicin. After a single injection of the compound in osteoarthritis (OA) patients, the pain of the patients was significantly reduced after 6 months [95]. Its efficacy in RA patients is not clear. These findings suggested that the role of TRPV1 in RA pain is very complex, and TRPV1 as a treatment target for RA pain may have unexpected consequences.

### 8.3. Acid-Sensing Ion Channels (ASICs)

ASICs are activated by endogenous lipids and reduce extracellular pH [96]. ASIC3 is very sensitive to changes of pH [97]. ASIC3 is expressed by synoviocytes, sensory fibers, and osteoclasts [50].

The expression of ASIC3 in the afferent nerve is increased in the chronic arthritis model [98]. The decrease of pH in arthritis may activate the ASIC3 channel expressed on DRG neurons which dominate the knee joint, thus transmitting pain signals to the spinal cord and brain and producing central sensitization [97]. In the ASIC3^−/−^ arthritis mouse model, synovial inflammation, bone erosion, and cartilage damage began to decrease from the 4th week, but bilateral hyperalgesia was decreased until the 6th week [92], indicating that ASIC3 is involved in chronic RA pain.

## 9. Conclusion

For patients with RA, pain may be their most serious problem, which will significantly affect their daily activities and work efficiency. Therefore, it is very important to find the appropriate treatment to alleviate the pain of RA patients. RA pain may be mediated by inflammation, peripheral sensitization, or the changes of central pain processing. How joint inflammation interacts with peripheral and CNS to cause persistent joint pain needs further study. It should be noted that cytokine candidates for CNS regulation mechanisms in RA pain may not be those traditionally involved in synovitis. With the in-depth study of the pain mechanism of RA, it is possible to find new regulatory factors and new therapeutic targets. In view of the central mechanism in the pain mechanism of rheumatoid arthritis, it is necessary to further study the efficacy of monoamine reuptake inhibitors, NMDAR antagonists, and other drugs for RA patients who still feel pain during the remission period. In addition, due to the overlapping function of multiple cytokines, a new strategy is to study the effect of bispecific antibodies against multiple cytokines on pain. Recently, it has been found that the bispecific antibodies targeting IL-1*β* and IL-17A have better effects on alleviating histological lesion and reducing the level of proinflammatory cytokines in CIA model than monovent IL-1*β* Mab or IL-17A Mab [99, 100]. However, the effect of bispecific antibody on RA pain has not been studied. Blocking multiple cytokines at the same time may provide synergistic or additive effects in pain relief. The causes of RA pain may be different in the early and late stages of the disease, during and between inflammatory attacks, and between different individuals. Therefore, individualized treatment programs need to be developed. This review will help us to further understand the mechanism of RA pain and open the door for more effective analgesic strategies.

## Figures and Tables

**Table 1:** After injecting different cytokines into normal knee joint, the changes of the responsiveness of the nociceptive sensory neurons (A*δ* and C fibers) to the mechanical stimulation of the joint. The effects of the intrathecal injection of cytokines on the responsiveness of the spinal cord induced by the joint mechanical stimulation and long-term effects of the neutralization of these cytokines on pain behavior in the animal model.

Cytokines	Responsiveness of A*δ* fibers to mechanical stim.	Responsiveness of C fibers to mechanical stim.	Responsiveness of the spinal cord to mechanical stim.	Effect of neutralization on mechanical hyperalgesia (route of administration)
TNF-*α*	Enhance slightly	Enhance	Enhance	Reduce (intra-articular/intrathecal)
IL-1*β*	Reduce	Enhance	Enhance	No effect (subcutaneous)
IL-6	No effect	Enhance (difficult to reverse)	Enhance (difficult to reverse)	Reduce (pretreatment is more effective than posttreatment; intra-articular application is more effective than systemic application)
IL-17	Reduce (at a very low dose of IL-17)	Enhance	Unknown	Reduce (intraperitoneal)
IL-22	Unknown	Unknown	Unknown	Reduce (i.a.)
*CXCL1 and CXCL2*	Unknown	Unknown	Enhance	Reduce (subcutaneous/intrathecal)
CX3CL1	—	—	Enhance	Reduce (intrathecal)

**Table 2:** Summarized studies showing the dual role of ion channels in RA pain.

Ion channels	Pharmacological interventions/transgenic animals	Effects	Reference
Ca^2+^*channels*	Blocking calcium channel	Reduce mechanical hyperalgesia	88, 89, 90
TRPV1	Neutralization of TRPV1	Reduce mechanical hyperalgesia	91
	TRPV1^−/−^ mice	Reduced late mechanical hyperalgesia	92
	Application of TRPV1 antagonists	Do not reduce mechanical hyperalgesia	94
ASIC3	ASIC3^−/−^ mice	Reduced late mechanical hyperalgesia	92
